# Ebola Virus Disease Outbreak — Democratic Republic of the Congo, August 2018–November 2019

**DOI:** 10.15585/mmwr.mm6850a3

**Published:** 2019-12-20

**Authors:** Aaron Aruna, Placide Mbala, Luigi Minikulu, Daniel Mukadi, Dorothée Bulemfu, Franck Edidi, Junior Bulabula, Gaston Tshapenda, Justus Nsio, Richard Kitenge, Gisèle Mbuyi, Celestin Mwanzembe, John Kombe, Léopold Lubula, Jean Christophe Shako, Mathias Mossoko, Felix Mulangu, Annie Mutombo, Emilia Sana, Yannick Tutu, Laetycia Kabange, Jonathan Makengo, Fortunat Tshibinkufua, Steve Ahuka-Mundeke, Jean-Jacques Muyembe, Ebola Response CDC, CDC Ebola Response, Walter Alarcon, Jesse Bonwitt, Dante Bugli, Nirma D. Bustamante, Mary Choi, Benjamin A. Dahl, Kevin DeCock, Amber Dismer, Reena Doshi, Christine Dubray, David Fitter, Margherita Ghiselli, Noemi Hall, Amen Ben Hamida, Andrea M. McCollum, John Neatherlin, Pratima L. Raghunathan, Fatima Ravat, Mary G. Reynolds, Adriana Rico, Nailah Smith, Gnakub Norbert Soke, Aimee T. Trudeau, Kerton R. Victory, Mary Claire Worrell

**Affiliations:** 1 Ministry of Health, Kinshasa, Democratic Republic of the Congo.; National Institute for Occupational Safety and Health, CDC; National Center for Emerging and Zoonotic Infectious Diseases, CDC; Center for Global Health, CDC; Center for Global Health, CDC; National Center for Emerging and Zoonotic Infectious Diseases, CDC; Center for Global Health, CDC; Center for Global Health, CDC; Center for Global Health, CDC; Center for Global Health, CDC; Center for Global Health, CDC; Center for Global Health, CDC; Center for Global Health, CDC; National Institute for Occupational Safety and Health, CDC; Center for Global Health, CDC; National Center for Emerging and Zoonotic Infectious Diseases, CDC; Center for Global Health, CDC; Center for Global Health, CDC; Center for Global Health, CDC; National Center for Emerging and Zoonotic Infectious Diseases, CDC; Center for Preparedness and Response, CDC; Center for Global Health, CDC; , Center for Global Health, CDC; National Center for Injury Prevention and Control, CDC; Center for Global Health, CDC; Center for Global Health, CDC.

On August 1, 2018, the Democratic Republic of the Congo Ministry of Health (DRC MoH) declared the tenth outbreak of Ebola virus disease (Ebola) in DRC, in the North Kivu province in eastern DRC on the border with Uganda, 8 days after another Ebola outbreak was declared over in northwest Équateur province. During mid- to late-July 2018, a cluster of 26 cases of acute hemorrhagic fever, including 20 deaths, was reported in North Kivu province.* Blood specimens from six patients hospitalized in the Mabalako health zone and sent to the Institut National de Recherche Biomédicale (National Biomedical Research Institute) in Kinshasa tested positive for Ebola virus. Genetic sequencing confirmed that the outbreaks in North Kivu and Équateur provinces were unrelated. From North Kivu province, the outbreak spread north to Ituri province, and south to South Kivu province (*1*). On July 17, 2019, the World Health Organization designated the North Kivu and Ituri outbreak a public health emergency of international concern, based on the geographic spread of the disease to Goma, the capital of North Kivu province, and to Uganda and the challenges to implementing prevention and control measures specific to this region (*2*). This report describes the outbreak in the North Kivu and Ituri provinces. As of November 17, 2019, a total of 3,296 Ebola cases and 2,196 (67%) deaths were reported, making this the second largest documented outbreak after the 2014–2016 epidemic in West Africa, which resulted in 28,600 cases and 11,325 deaths.^†^ Since August 2018, DRC MoH has been collaborating with partners, including the World Health Organization, the United Nations Children’s Fund, the United Nations Office for the Coordination of Humanitarian Affairs, the International Organization of Migration, The Alliance for International Medical Action (ALIMA), Médecins Sans Frontières, DRC Red Cross National Society, and CDC, to control the outbreak. Enhanced communication and effective community engagement, timing of interventions during periods of relative stability, and intensive training of local residents to manage response activities with periodic supervision by national and international personnel are needed to end the outbreak.

## Epidemiology and Laboratory Testing

After declaration of the outbreak on August 1, 2018, rapid response teams that included clinicians, epidemiologists, and local public health officials were deployed to health zones in North Kivu, South Kivu, and Ituri provinces. The response teams interviewed patients and household contacts to identify secondary cases and contacts. Teams used standardized case investigation forms to classify cases as suspected, probable, or confirmed during August 1, 2018–November 17, 2019. A suspected case (in a person who was living or had died) was defined as the acute onset of fever (≥100°F [≥38°C]) and at least three Ebola-compatible clinical signs or symptoms (headache, vomiting, anorexia, diarrhea, lethargy, stomach pain, muscle or joint aches, difficulty swallowing or breathing, hiccups, unexplained bleeding, or any sudden, unexplained death) in a North Kivu, South Kivu, or Ituri resident or any person who had traveled to these provinces during this period and reported signs or symptoms defined above. A patient who met the suspected case definition who had died and from whom no specimens were available was considered to have a probable case. A confirmed Ebola case was defined as a suspected case with at least one positive test for Ebola virus using reverse transcription–polymerase chain reaction (RT-PCR) (*3*) testing. Patients with suspected Ebola were isolated and transported to an Ebola treatment center for confirmatory testing and treatment. Oral swabs were collected from decedents with suspected cases within 24 hours of notification of death and sent to a DRC laboratory for confirmation of Ebola virus. All eight DRC laboratories have Ebola virus diagnostic capacity using GeneXpert (under emergency use authorization) as the primary diagnostic RT-PCR test for qualitative detection of Zaire ebolavirus RNA (*1*).

**FIGURE 1:**
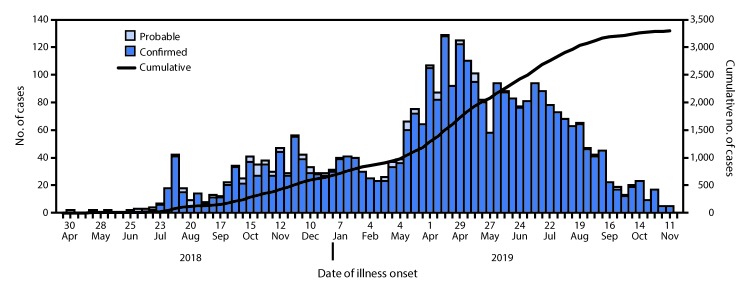
Confirmed and probable cases of Ebola virus disease by week of illness onset and cumulative number of cases — Democratic Republic of the Congo, April 30, 2018–November 17, 2019

**FIGURE 2:**
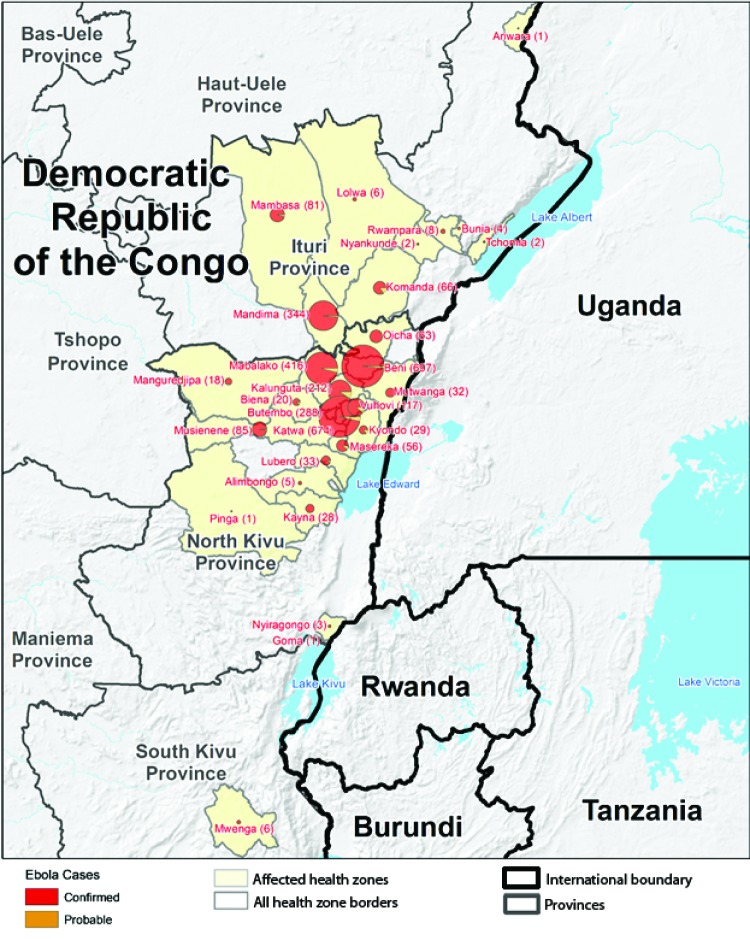
Geographic distribution of confirmed and probable cases of Ebola virus disease (Ebola) by health zones — North Kivu, South Kivu, and Ituri Provinces, Democratic Republic of the Congo, April 30, 2018–November 17, 2019* * During April 30, 2018–November 17, 2019, a total of 3,296 Ebola cases (3,178 confirmed and 118 probable) were reported by the Democratic Republic of the Congo (DRC) Ministry of Health. In addition, three persons in Uganda who had traveled from Uganda to DRC to attend the funeral of a DRC Ebola patient became infected and died.

During April 30, 2018–November 17, 2019, a total of 3,296 Ebola cases (3,178 confirmed and 118 probable) (Figure 1) and 2,196 (67%) deaths were reported by DRC MoH. The five most affected health zones were Beni (697 cases), Katwa (674), Mabalako (416), and Butembo (288) in North Kivu Province and Mandima (344) in Ituri Province. These five health zones accounted for 69% of all cases reported to date (Figure 2). A majority of cases (1,857, 56%) occurred in females, and 968 (29%) occurred in persons aged ≤18 years. Health care workers accounted for 163 (5%) cases. Thirty-four percent of cases were community deaths (i.e., Ebola cases not identified until patient death and thus not effectively isolated from the time of infection until death). As of November 17, 2019, approximately 1,492 (45%) of the 3,296 cases and 150,000 contacts of patients with confirmed and probable Ebola had been monitored across all affected health zones for 21 days after their last known exposure. However, contact enumeration was incomplete because insecurity caused by conflict, mistrust toward local authorities, and resistance prevented rapid response teams from entering some communities.

Conflict, including clashes between armed groups and Congolese security forces, has resulted in eruptions of violence targeting civilians and displacement of tens of thousands of residents into neighboring provinces and countries (Rwanda and Uganda). On June 11, 2019, the Uganda Ministry of Health reported a patient with confirmed Ebola who had traveled to DRC for a funeral and then back to Kasese district in eastern Uganda. The patient was a child aged 5 years who had traveled with five family members from Uganda to DRC to attend the funeral of his grandfather, who had died from probable Ebola. The day after the funeral, two additional family members who had traveled from Uganda to DRC were confirmed to have Ebola. All three Ebola patients died after returning to Uganda, and no additional cases have been reported in Uganda since June 12, 2019. The confirmed cases in Uganda are the first cases of Zaire ebolavirus infection in that country and the first cases reported in Uganda since 2013.

On July 14, 2019, a confirmed case of Ebola was reported in a traveler to Goma, a city in DRC with a population of >1 million that is located on the border with Rwanda. The patient traveled by bus from Butembo approximately 190 miles (300 km) north of Goma and died on July 16, 2019; contact enumeration is complete, and 21-day follow-up has been completed. This case was the first reported in a major urban center in the current outbreak, prompting an intensification of response efforts. On July 30, 2019, another confirmed case was also reported in Goma. The patient, who traveled by bus from a community near Bunia in Ituri province, approximately 350 miles (560 km) north of Goma, died at Goma’s Ebola treatment center on July 31, 2019. In addition, two secondary confirmed cases in family members who were contacts of the patient received medical care in Goma’s Ebola treatment center and contact enumeration has been completed for this transmission chain.

## Public Health Response

DRC MoH has established a strategic coordination center in Goma, with an emergency operations center (EOC) that monitors both implementation of the operations through lower administrative level EOCs that report to Goma and direct contacts with the teams in the health zones. In addition, the EOC and DRC MoH commissions (e.g., surveillance, vaccination, and safe and dignified burials) also coordinate the deployment of multidisciplinary rapid response teams to support affected health zones.

Since August 1, 2018, DRC MoH has been collaborating with several international partners to support response activities and enhance Ebola preparedness. To strengthen surveillance activities, DRC MoH disseminated standardized Ebola case definitions, developed reporting tools and communication strategies, and began distribution of daily situation reports. Rapid response teams have deployed to affected health zones to strengthen Ebola case management and infection prevention and control in health care facilities and in 14 treatment and transit centers. An experimental single-dose Ebola vaccine licensed by Merck (recombinant vesicular stomatitis virus–Zaire Ebola virus [rVSV-ZEBOV-GP]) (*4*) has been authorized under compassionate use by the World Health Organization and DRC MoH. The vaccine is provided primarily through a ring vaccination strategy that targets contacts of index cases and their contacts. The vaccine is also offered to groups at high risk, such as health care personnel and frontline workers (those whose duties [e.g., case investigation, burial, or vaccination] puts them at high risk for Ebola infection). As of November 17, 2019, approximately 250,000 persons at risk for Ebola have been vaccinated, including approximately 31,000 health care and frontline workers. In addition, regulatory authorities in DRC have approved the use of four therapeutic agents that have been effective in nonhuman primates for compassionate use in patients with Ebola; these include the monoclonal antibodies MAb114, REGN-EB3, ZMapp, and the antiviral remdesivir. The effectiveness of these therapeutic agents was evaluated in a trial using an Ebola virus generated via a reverse genetics system and Ebola virus sequences provided by organizations in DRC (*5*). Preliminary results from the study led the trial’s monitoring board to stop the study and randomize all remaining patients to either mAb114 or REGN-EB3 because both of these agents were found to decrease case fatality rates (*5*).

## Discussion

The first human Ebola outbreak occurred in Zaire (now DRC) in 1976, and since then approximately 28 known outbreaks of Ebola have occurred in Africa (*6*). Although DRC has successfully contained Ebola outbreaks in the past (*4*,*6*), challenges specific to North Kivu and Ituri provinces have complicated the current outbreak control. Limited infrastructure coupled with armed conflict among rebel groups, DRC’s armed forces, and militants attacking civilians have led to insecurity resulting in interruptions in response activities (*2*,*7*). The prolonged conflict has seeded mistrust toward local authorities and international partners, which has impeded effective community collaboration and led to incomplete case ascertainment and contact enumeration, vaccination refusals, and delayed seeking of health care. Nosocomial transmission of disease in local health facilities has further eroded communities’ confidence in the health system (*2*,*5*). Hesitant patients have absconded from Ebola treatment centers, and families have resisted taking patients to hospitals, thereby increasing disease transmission in communities. In addition, contact with an infected corpse or body fluids of an infected person, especially after a community death of a patient with suspected Ebola or during unsafe burials (*8*,*9*) has increased community transmission. Intervention strategies to decrease community concerns regarding Ebola intervention measures, such as involvement of local leaders and health education, have been successful and need to be continued to reduce Ebola virus transmission in communities (*2*,*9*). These strategies include 1) educating residents about the signs and symptoms of Ebola and its modes of transmission, 2) emphasizing the importance of seeking medical care and promptly reporting suspected Ebola cases, 3) emphasizing the potential benefit of early diagnosis and treatment with effective Ebola therapeutics (*5*), and 4) trusted local leaders disseminating health communication messages in local languages. These steps can facilitate the isolation and treatment of patients in a reserved ward in local hospitals or in the homes of patients unwilling to seek care at an Ebola treatment center (*9*).

Shortage of response personnel and ongoing strain on limited resources are important issues that need to be addressed to improve data management for the response at the national level. The work of the EOC has improved the ability of DRC MoH to respond to this epidemic and identify targeted intervention strategies for affected health zones. Compared with earlier outbreaks, this outbreak is occurring in a context of armed conflict, and innovative approaches beyond the conventional Ebola response are needed (*10*). These approaches include the building of trust with communities amid insecurity, opportunistically timed intensive interventions during periods of relative stability, and intensive training of local residents to manage response activities, with periodic supervision by national and international personnel as a public health priority.

**Summary**

**What is already known about this topic?**

The Democratic Republic of the Congo (DRC) is currently experiencing its tenth outbreak of Ebola virus disease (Ebola), which was designated a public health emergency of international concern by the World Health Organization on July 17, 2019.

**What is added by this report?**

As of November 17, 2019, a total of 3,296 Ebola cases and 2,196 (67%) deaths have been reported. Challenges to outbreak control include armed conflict between rebel groups and DRC’s armed forces, which has interrupted response activities, and community mistrust.

**What are the implications for public health practice?**

Enhanced communication and effective community engagement, timing of interventions during periods of relative stability, and intensive training of local residents to manage response activities with periodic supervision by national and international personnel would help end the outbreak sooner.

## Footnotes

* https://www.cdc.gov/vhf/ebola/history/2014-2016-outbreak/index.html.

† https://apps.who.int/iris/bitstream/handle/10665/273640/SITREP_EVD_DRC_20180807-eng.pdf?ua=1.
